# Prevention and Inhibition of TC-1 Cell Growth in Tumor Bearing Mice by HPV16 E7 Protein in Fusion with Shiga Toxin B-Subunit from *shigella dysenteriae*

**Published:** 2013-07-02

**Authors:** Mohammad Sadraeian, Mohammad Javad Khoshnood Mansoorkhani, Milad Mohkam, Sara Rasoul-Amini, Mahdi Hesaraki, Younes Ghasemi

**Affiliations:** 1 Pharmaceutical Sciences Research Center, School of Pharmacy, Shiraz University of Medical Sciences, Shiraz, Iran; 2 Department of Pharmacology and Toxicology, School of Pharmacy, Shiraz University of Medical Science, Shiraz, Iran; 3 Department of Pharmaceutical Biotechnology, School of Pharmacy, Shiraz University of Medical Science, Shiraz, Iran; 4 Department of Medicinal Chemistry, School of Pharmacy, Shiraz University of Medical Sciences, Shiraz, Iran; 5 Department of Stem Cells and Developmental Biology at Cell Science Research Center, Royan Institute for Stem Cell Biology and Technology, ACECR, Tehran, Iran

**Keywords:** Protein Vaccine, E7-STxB, Immunization, Tumor Growth, Cervical Cancer

## Abstract

**Objective:**

For immunotherapy of human papillomavirus (HPV) -16-associated cervical cancers the E7 protein is considered a prime candidate. However it is a poor inducer of cytotoxic T-cell response, when being used as a singular antigen in protein vaccination. Hence, in this study we focused on the utilization of a vaccine delivery system for prevention or treatment of cervical cancer.

**Materials and Methods:**

In this experimental study, we designed and evaluated a novel fusion protein comprising HPV16 E7 antigen fused to Shiga toxin B-subunit (STxB) as both an antigen vector and an adjuvant. Then we designed two preventive and therapeutic tumor models to investigate the prevention and inhibition of TC-1 cell growth in female C57BL/6 mice, respectively. In each model, mice were immunized with the recombinant protein of E7-STxB or E7 without any adjuvant.

**Results:**

We demonstrated that prophylactic immunization of E7-STxB protected mice against TC-1 cells. Also in the therapeutic model, E7-STxB inhibited TC-1 tumor growth inlungs. The results were significant when compared with the immunization of E7 singularly.

**Conclusion:**

We concluded that immunization with the E7-STxB protein without any adjuvant could generate anti-tumor effect in mice challenged with TC-1 cells.This research verifies the clinical applications and the future prospects of developing HPV16 E7 therapeutic vaccines fused to immunoadjuvants.

## Introduction

Cervical cancer is the second most common cause of cancer-related deaths in women worldwide. Human papillomavirus (HPV) is the most prevalent, accounting for more than half of cervical cancer cases. The HPV oncogenic proteins, E6 and E7, are important in the induction and maintenance of cellular transformation and co-expressed in most HPV-containing cervical cancers (1-4). Vaccines or immunological therapeutics targeting E7 and/or E6 proteins may provide an opportunity to treat HPV-associated cervical malignancy (5-8). Therefore, in this study, human HPV-16 E7 was chosen for vaccine development.

In order to overcome many of the disadvantages of peptide vaccines, using of full-size proteins as model antigens can be considered. As the antigen cannot be introduced into the MHC class I antigen presentation pathway, the administration of soluble proteins alone mostly may not induce cytotoxic T lymphocytes (CTL) responses. Hence their efficiency to elicit antibody is not also often comparable, requiring the use of adjuvant (9, 10). Thus in most cases peptide vaccines failed to elicit effective immune and clinical responses (11).

One the other hand, Shiga toxin from Shigella dysenteriae is composed of an A subunit, which mediates toxicity, and a B subunit (StxB), a nontoxichomopentameric protein responsible for toxin binding and internalization into target cells by interacting with the glycolipid globotriaosylceramide (Gb3 or CD77) (12) which is almost exclusively expressed on cancer cells, dendritic cells (DC) and B cells (13, 14).

As STxB can efficiently target peptides into the MHC class I pathway and induced peptidespecific CTL in mice, it has the potential to act as both an antigen vector and an adjuvant in enhancing antigen-specific tumor immunity. So it is tempting to propose the use of STxB for tumor cell delivery purposes (15). In this study, we investigated the prevention and inhibition of TC-1 cell growth as a model of cervical cancer (16) by using the soluble E7-STxB compared with the sole E7 protein expressed in the host *E. coli* BL21 (DE3). This study focuses on the utilization of a vaccine delivery system for prevention or treatment of cervical cancer.

## Materials and Methods

### Chemical reagents (enzymes, vectors, bacterial strains, recombinant proteins)

Pfu and taq DNA polymerase (2.5 U/μl, Fermentas, Lithuania), enzymes NdeI, SalI and NotI (Fermentas, Lithuania.), IPTG (Vivantis, Malaysia), Vector pET-28a (+) (Novagen USA), Vector pGEM-T (Promega, USA), and stxB gene from *shigella dysenteriae* type I (Imam Hossein University, Tehran, Iran) was prepared. Following the method we previously described (17, 18), and used as the template in PCR experiment. *E. coli* DH5α and *E. coli* BL21 (DE3) was used for cloning and expression experiments. Plasmid pGEM-T Vector and pET-28a (+) were used as cloning and expression vectors respectively.

### Cell lines

TC-1 cells expressing HPV16-E6 and HPV16- E7 proteins were purchased from a cell bank (Pasteur Institute of Iran). The tumor cell line, TC-1, was derived from primary lung epithelial cells of C57BL/6 mice. The cells were immortalized with the amphotropic retrovirus vector LXSN16E6E7 and subsequently transformed with the pVEJB plasmid expressing the activated human c-Ha-ras oncogene (19).They were cultured in RPMI 1640 (PAA, Austria) supplemented with 10% heat-inactivated fetal calf serum, insulin, growth factor, 2 mM L-glutamine, 1 mM pyruvate, 0.1mM minimal essential medium with nonessential amino acids, 100U penicillin/ml and 100 μg streptomycin/ ml, and was incubated at 37˚C in 5% CO_2_.

### Mice

Six to seven week-old female C57BL/6 mice were obtained from Animal Lab, Shiraz University of Medical Sciences, Iran. Given free access to food and water, the mice were housed for one week before the experiment, and were maintained at standard condition. All experiments were done in accordance with the Animal Care and Usage Protocol of Shiraz University of Medical Sciences, Iran.

### Plasmids construction

**Fig 1:**
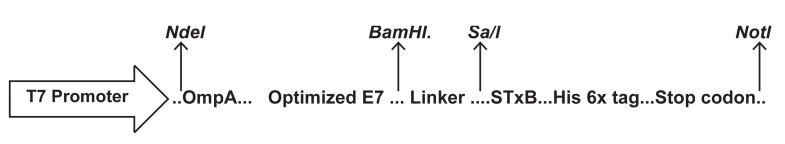
Schematic representation of the fusion construct employed for *E. coli* expression in pGH vector. Outer membrane protein A (ompA) and Histidine tag are indicated.

As previously described (20), the amplified stxB gene fragment (207 bp). from *shigella dysenteriae* type I and synthetic codon optimized HPV16 E7 were fused as E7-STxB chimeric gene (Fig 1). The amplified fused fragments were cloned in pGEM vector and transformed into *E. coli* DH5α. The E7-stxB fragment was subcloned into indigested pET28a (+) as an expression vector and to construct pET28a (+)-E7-stxB. Separately, the amplified E7 gene was subcloned into undigested pET28a (+) vector to construct pET28a (+)-E7. Subsequently, the pET-28a (+)-E7-stxBwas confirmed by PCR and restriction enzyme digestion.

### Protein production, purification and characterization of E7 and E7-STxB proteins

Expression of E7 (13 kDa) and E7-STxB (28kDa) proteins followed the procedure we previously described (20). The E7 and E7- STxB proteins were expressed efficiently in *E. coli* BL21 (DE3) after 4 hours of induction by isopropyl-β-d-thiogalactoside (IPTG). Inclusion body and supernatant were analyzed by SDS-PAGE ona 12% gel. These proteins were expressed as inclusion bodies. The inclusion body that contained the E7 or E7-STxB protein was then sonicated and washed twice with 100 ml solution (0.5 M NaCl, 20 mMTris, 2 M urea, 0.5% Triton, pH=7.9) and centrifuged at 8000 rpm for 20 minutes at 4˚C. The resultant pellet was resuspended in 30 ml buffer solution (0.5 M NaCl, 20 mMTris, 8 M urea, pH=7.9), sonicated and centrifuged at 16,000 rpm for 20 minutes at 4˚C. The supernatant (soluble protein) was dialyzed three times in 1l solution (0.5 M NaCl, 20 mMTris, 3 M urea, pH=7.9) at 4˚C for 1 hour. Subsequently, the protein solution was dialyzed extensively with PBS at 4˚C and the final supernatant (soluble protein solution) was obtained by centrifuging at 16,000 rpm for 20 minutes at 4˚C. Removal of contaminating LPS was conducted using ToxinEraserTM Endotoxin Removal Resin (GenScript USA Inc.)

The identity and the purity of the recombinant proteins were determined by SDS-PAGE. Concentrations of proteins were measured by the Bradford assay. To confirm the characteristics of the recombinant proteins, human HPV16 E7 antibody (Santa Cruz Biotechnology, Santa Cruz, CA, USA) were used to verify all the purified proteins by western blot analysis.

### Prevention of TC-1 cell growth by immunization with E7 or E7-STxB protein

In the preventive tumor model, 12 female C57BL/6 mice from both the control and the experiment group were immunized subcutaneously with 1.5 nmol of either E7 or E7-STxB protein in 100 μl PBS as experiment groups and also with only 100 μl PBS as the control group. A second equivalent dose of the same protein was given by intraperitoneal injection two weeks later. The mice were again injected subcutaneously with 1×10^5^ TC-1 cells in the right flank seven days after the second immunization. After 90 days, tumor growth was monitored and tumor incidence was also recorded.

### Inhibition of TC-1 cell growth with E7 or E7- STxB protein

In therapeutic tumor model, 24 mice were injected intravenously into thetail vein with 1×10^5^ TC-1 cells, and then immunized subcutaneously with 1.5 nmol of protein on the next day. The model consisted of three groups with eight mice in each. The groups were divided into two experiment groups of E7 and E7-STxB, and one control group of PBS. One week later, mice were immunized by intraperitoneal injection with the same dose of protein. Starting 7-10 days later and every 3-4 days there after, the area was observed and palpated for the presence of a tumor nodule. Four weeks later, six in each group were randomly by CO_2_ inhalation. The lungs were resected and inspected for tumor growth using anatomical dissecting microscopy with ×2 magnification. The number of tumor nodules on lungs was counted and the weight of the body and lungs were recorded. Subsequently, the average mean of all tumor sizes were calculated and reported in millimeters. Tumor diameters were measured in two orthogonal dimensions using electronic digital calipers. Tumor volumes were calculated from these measurements according to: (length×width^2^)/2. Tumor sizes (in millimeters) were reported as the average of all measured dimensions.

### Statistical analysis

All data expressed as means ± SD are representative of two to four different experiments. All the statistical analyses were analyzed by oneway ANOVA followed by Tukey’s test. In all cases, the differences showing (p<0.05) were considered as significant.

## Results

### Characterization of constructs

**Fig 2:**
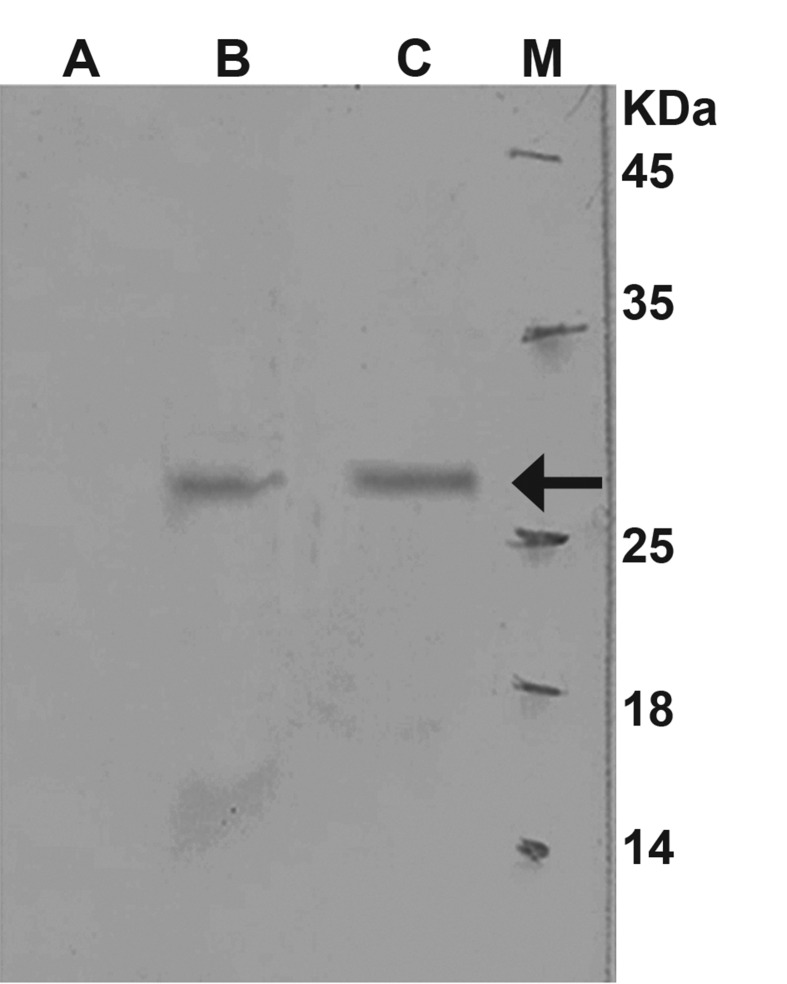
Western blot analysis of secreted E7-STB-His6 into supernatant. (M) protein molecular marker; (A) Soluble lysate of cells without recombinant plasmid as negative control; (B) 5 hours after induction at 37 ˚C; (C) 22 hours after induction at 22 ˚C. The bands are shown by arrows.

The E7 and E7-STxB proteins were characterized by SDS-PAGE electrophoresis and subsequent western blot analysis (Fig 2). The purified proteins were observed by SDSPAGE and the amount of protein was calculated by Bradford protein assay used for mice injection. The percentage of soluble recombinant protein yield of total cell protein was 36% under optimized conditions.

### Immunization with E7-STxB prevents the TC-1 growth

A preventive tumor model was used to test the anti-tumor immunity of E7-STxB protein. Female C57BL/6 mice were immunized twice with soluble E7-STxB or E7 protein. After being challenged with 1×10^5^ TC-1 cells, all the mice of the control group who were immunized with only 100 μl PBS developed tumors, whereas no tumor was developed in all the mice immunized with E7-STxB protein for over 90 days. The percentage of tumor incidence for the mice immunized with E7 protein was not significant in comparison with the control group.

### E7-STxB elicits stronger inhibition of TC-1 growth

**Fig 3:**
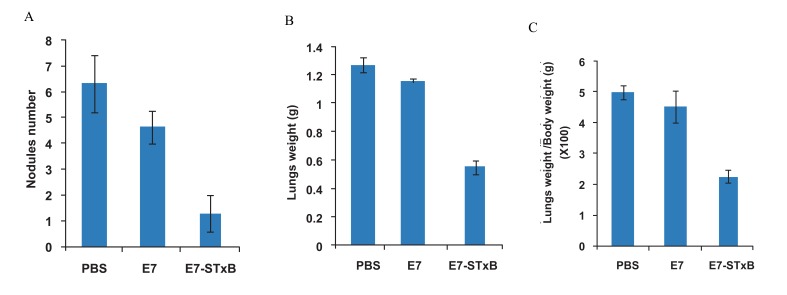
In therapeutic model, four weeks after last injection, six mice in each group were euthanized randomly by CO_2_ inhalation. (A); The number of tumor nodules on lungs was counted. (B); The body and lungs weights were recorded. (C); Value of Lungs weight/Body weight ×100 was calculated.

**Fig 4:**
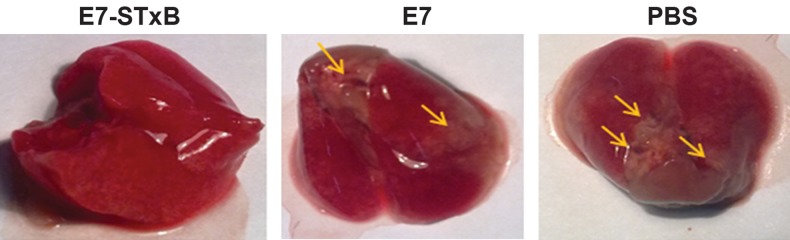
In therapeutic model, four weeks after last injection, six mice in each group were euthanized randomly by CO_2_ inhalation. (A); The number of tumor nodules on lungs was counted. (B); The body and lungs weights were recorded. (C); Value of Lungs weight/Body weight ×100 was calculated.

In the therapeutic tumor model, four weeks after the last injection, six mice of each group were sacrificed randomly and the number of tumor nodules on lungs was counted (Fig 3A). In figure 4, the lungs of the three groups areshown with arrows indicating the tumor nodules. The number of tumor nodules was 6.3 ± 1.1 in the PBS group, 4.5 ± 0.6 in the E7 group and 1.3 ± 0.7 in the E7-STxB group. The weight of the body and lungs were then recorded. The weight of the lungs was 1.271 ± 0.036 g in the PBS group, 1.164 ± 0.131 g in the E7 group, and 0.554 ± 0.047 g in the E7- STxB group (Fig 3B). The value of Lungs weight/body weight ×100 was calculated. It was 5.019 ± 0.210, 4.523 ± 0.515 and 2.279 ± 0.176 in the PBS, E7 and E7-STxB groups respectively (Fig 3C).

### Analysis of the tumor sizes

The volume of tumors was computed using the formula: V=(a^2^b)/2. It was 624.3 ± 62.6 in the PBS group, 182.4 ± 41.3 in the E7 group and 125.3 ± 0.8 in the E7-STxB group. There were significant decreases in tumors sizes and improvements in the mice treated with E7 (p<0.001) and E7-STxB (p<0.001) compared to PBS only. The mean tumor volumes and tumor sizes were not significantly different between the E7and the E7-STxB groups (p=0.09).

## Discussion

The HPV 16 E7 protein is regarded as a prime candidate in developing a therapeutic vaccination against human papillomavirus-related diseases. Unfortunately, the experimental use of HPV 16 E7 protein in a simple vaccination has been proven to be rather inefficient in inducing a potent CTL response. Therefore, different approaches have been undertaken to improve the immunogenicity of E7 in protein vaccination. Among them is fusion with heat shock protein or other immunoadjuvants and directing the protein into the MHC class I pathway and inducing peptide-specific CTL (13-15).

STxB has been reported to have the potential to act as both an antigen vector and an adjuvant in enhancing antigen-specific tumor immunity (13, 14), however, there are some controversy regarding this matter (11, 13). Hence, in order to induce and expand regulatory T-cells with immunosuppressive functions, attaining the soluble form of STxB-based vaccine seems necessary (15, 20). Most of the fusion proteins, like antigens fused to STxB, are found in inclusion bodies, and therefore, have to be refolded after denaturation with urea.

In this study, not only immunization but also protection from the tumor challenge was tested in C57BL/6 mice by using the tumor cell line TC-1, which expresses HPV16 E7. In the immunization model, we demonstrated that the injection of E7-STxB in soluble form protected the mice from tumor challenge completely, even in the absence of adjuvant. Subsequently, in the therapeutic model, the difference between the number of tumor nodules in control PBS group and E7-STxB group was statistically significant (p<0.001), but no significant difference between PBS group and E7 group was observed. Also there was significant difference between E7-STxB and E7 group (p<0.05).

The value of Lungs weight/Body weight ×100 showed that there was significant difference between E7-STxB and the PBS or the E7 group (p<0.001). The difference between the E7 group and the PBS group showed a p value of 0.055. These data suggested that E7-STxB immunization could elicit stronger inhibition of TC-1 growth on lungs compared to E7 immunization. These findings raise the possibility that STxB may be an efficient agent in generating cell mediated immune responses. Therefore, the STxB protein may increase the therapeutic potential of E7 protein-based vaccine against cervical cancer in women.

## Conclusion

Immunization with E7-STxB protein without any adjuvant could produce efficient anti-tumor effect in mice challenged with TC-1 cells, compared to E7 only-based immunization. This study verifies the clinical applications and the future prospects of developing HPV16 E7 therapeutic vaccines fused to immunoadjuvants.
